# Prediction of Novel Natural Small Molecules From Schinus molle as an Inhibitor of PI3K Protein Target in Cancer Cells Using In Silico Screening

**DOI:** 10.7759/cureus.50863

**Published:** 2023-12-20

**Authors:** Umesh Kumar, Hema Priya Manivannan, Arul Prakash Francis, Vishnu Priya Veeraraghavan, Gayathri R, Kavitha Sankaran

**Affiliations:** 1 Centre of Molecular Medicine and Diagnostics (COMManD) Department of Biochemistry, Saveetha Dental College and Hospitals, Saveetha Institute of Medical and Technical Sciences, Saveetha University, Chennai, IND

**Keywords:** pi3k, novel compounds, therapeutics, schinus molle, mortality, cancer

## Abstract

Introduction

Cancer continues to pose a significant challenge in medical research. Phytochemicals derived from plants have emerged as a promising avenue for pioneering drug discovery due to their potential for reduced toxicity. The phosphatidylinositol 3-kinase (PI3K) pathway has gained recognition as a pivotal signaling pathway with implications across multiple facets of cancer initiation and progression. This study focuses on the virtual screening of phytochemicals from *Schinus molle*, evaluating their potential as inhibitors of PI3K, a crucial target in cancer therapy.

Methods and materials

The present study involved a comprehensive in silico screening of phytochemicals derived from *S. molle. *The screening process encompassed various parameters, such as drug-likeness, pharmacokinetics, molecular docking, toxicity analysis, bioavailability assessment, and molecular target exploration. The primary objective of this systematic approach was to identify potential lead compounds. The study aimed to provide a detailed understanding of the molecular properties of the phytochemicals and their potential as drug candidates.

Results

Upon analyzing 18 compounds, two compounds were noteworthy. Beta-spathulene and kaempferol demonstrated significant affinity for PI3K and favorable attributes concerning drug-likeness, pharmacokinetics, and bioavailability.

Conclusion

While our computational investigation lays a promising foundation, it is essential to emphasize that further experimental studies, including* in vitro* and *in vivo *experiments, are imperative to validate the action of these lead compounds.

## Introduction

Cancer remains one of the most formidable challenges in the field of medical research, affecting millions of lives globally [1,2]. The intricate nature of cancer's molecular mechanisms demands innovative therapeutic strategies to combat its progression [3]. The phosphatidylinositol 3-kinase (PI3K) pathway has emerged as a critical signaling pathway in cancer development and progression [4]. Dysregulation of the PI3K pathway is frequently observed in many cancers, making it an attractive target for therapeutic interventions [5].

Natural products have garnered significant attention recently as potential sources of novel drug candidates due to their diverse characteristics and often favorable pharmacological properties [6]. *Schinus molle*, commonly known as the Peruvian pepper tree, is a plant with a rich history of traditional medicinal use in various cultures [7,8]. This plant is known to harbor an array of bioactive compounds, some of which have shown promising biological activities, including anti-inflammatory, antioxidant, and antimicrobial properties [9-11].

In the pursuit of identifying novel therapeutic agents for targeting the PI3K pathway in cancer, computational approaches have gained prominence [12,13].* In silico* screening involves computer-based tools and algorithms that have proven invaluable in identifying and characterizing potential small molecules as inhibitors of specific molecular targets [14-16]. These approaches allow for the rapid screening of vast chemical libraries, enabling researchers to predict the binding interactions between a potential inhibitor and its target protein [17].

This research aims to employ *in silico* methodologies to investigate the viability of phytochemicals from *S. molle* as potential inhibitors targeting the PI3K pathway in cancer. Phytochemicals possessing characteristics of druglikeness and pharmacokinetics properties were subjected to molecular docking with the target PI3K. Compounds with favorable affinity were then subjected to toxicity analysis. In addition, the compounds will be scrutinized for their bioavailability and molecular target interactions.

## Materials and methods

Ligands and proteins

Active compounds from *S. molle* were obtained from Dr. Dukes’ Phytochemical and Ethnobotanical Databases [18]. The PubChem database was employed to access the phytochemicals’ structures (2D and 3D) and canonical simplified molecular-input line-entry system (SMILES) representations. Compounds not present in PubChem were excluded from the study [19]. As a comparative reference, the control drug copanlisib was included. Initially, 18 compounds underwent preliminary screening before being chosen for a more comprehensive analysis. The 3D structure of PI3K was retrieved from the RCSB Protein Data Bank (PDB) and identified by the proteins’ PDB ID 4FA6 [20]. 

Drug-likeness and pharmacokinetics

All 18 compounds and the control drug were evaluated for their drug-likeness and pharmacokinetic properties. This evaluation was conducted by submitting the compounds’ SMILES representations to the SwissADME online web server [21]. The screening process adhered to Lipinski’s criteria, encompassing parameters, such as a molecular weight below 500 Da, hydrogen bond donors of fewer than five, hydrogen bond acceptors of fewer than 10, and a log P value of less than 5. These criteria were employed to gauge oral bioavailability and drug-likeness.

Furthermore, the compounds’ pharmacokinetic attributes were evaluated, encompassing factors, such as gastrointestinal tract (GIA) absorption, permeability across the blood-brain barrier (BBB), potential as a p-glycoprotein substrate, and interactions with cytochrome P450 isoenzymes.

Molecular docking

Molecular docking is an essential technique in novel drug development, as it plays a pivotal role in predicting the binding affinity between a ligand and its specific target. In the present study, the target protein, PI3K, was subjected to molecular docking with phytochemicals possessing favorable drug-likeness and pharmacokinetic characteristics and the control compound. The molecular docking simulations were conducted using AutodockTools 1.5.7 (The Scripps Research Institute, USA), following the methodology outlined by Roy et al. [22].

The preparation of the PI3K protein involved removing water molecules and adding hydrogen bonds, along with the computation of Gasteiger charges. The ligands were granted flexibility during the docking process. Grid coordinates were established based on the binding site of the existing inhibitor within the receptor. From the array of resulting conformations, the one exhibiting the least binding affinity was chosen for further analysis. The visual representation of the docked pose was accomplished using BIOVIA Discovery Studio Visualizer v21.1.0.20298 (Dassault Systemes, France).

Toxicity analysis

Compounds exhibiting binding affinities within the range of -6.5 to -8 kcal/mol underwent scrutiny for potential organ toxicity, particularly hepatotoxicity. Other critical toxicological factors, including carcinogenicity, immunogenicity, mutagenicity, and cytotoxicity, were also anticipated using the online platform ProTox-II [23]. Those compounds that demonstrated no predicted toxicity were considered lead candidates and subjected to an in-depth analysis.

Bioavailability radar and molecular target prediction

After identifying the promising lead compounds, an extended evaluation was conducted to ascertain their bioavailability using the SwissADME web server. This comprehensive analysis aimed to provide insights into how efficiently lead compounds could be absorbed, distributed, metabolized, and excreted within a biological system.

In addition to assessing bioavailability, the Swiss target prediction web server was utilized to delve into the potential molecular interactions of the identified lead compounds [24]. This step involved predicting the specific protein targets within the biological system that these lead compounds could potentially bind.

## Results

Ligand and protein structure

**Figure 1:**
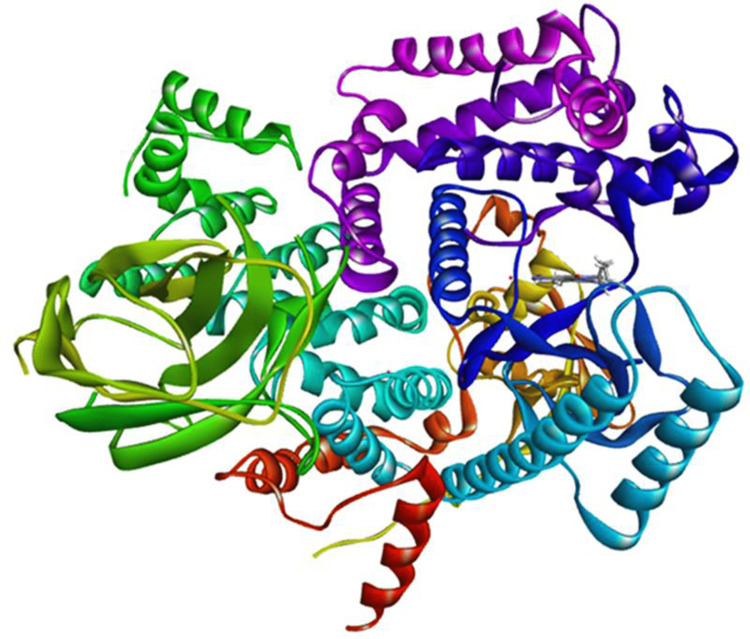
3D structure of the target protein

The list of phytochemicals derived from *S. molle* is presented in Table 1. Furthermore, Figure 1 provides a graphical representation of the 3D structure of the PI3K target derived from the PDB.

**Table 1:** Phytochemicals of Schinus molle selected for the screening studies

S. No.	Compound
1	Alpha-pinene
2	Beta-phellandrene
3	Beta-pinene
4	Beta-sitosterol
5	Beta-spathulene
6	Camphene
7	Carvacrol
8	Caryophyllene
9	D-Alpha-phellandrene
10	D-Limonene
11	Fisetin
12	Gallic-acid
13	Kaempferol
14	Myrcene
15	Myricetin
16	Perillaldehyde
17	Quercetin
18	Sobrerol
C	Copanlisib

Drug-likeness and pharmacokinetics

In this study, compounds that exhibited a single violation of Lipinski's rule were deemed acceptable for further analysis. Notably, none of the 18 compounds examined demonstrated any violations of Lipinski's rule of five, indicating their favorable molecular characteristics regarding drug-likeness.

The bioavailability evaluation was gauged through a bioavailability score, with all compounds yielding a score of 0.55. It was found that gallic acid exhibited a slightly elevated bioavailability score of 0.56. This suggests that these compounds possess effective absorption and distribution within a biological system, enhancing their potential as drug candidates.

Table 2 provides a detailed presentation of the drug-likeness of phytochemicals. This analysis sheds light on the suitability of phytochemicals for further exploration and potential utilization in drug development efforts.

**Table 2:** Drug-likeness of phytochemicals in Schinus molle Mol. weight: molecular weight; NHBD: number of hydrogen bond donor; NHBA: number of hydrogen bond acceptor; RB: rotatable bond; TPSA: topological polar surface area

S. No.	Phytochemicals	Mol. weight	NHBD	NHBA	Rotatable bond	Log P	Molar refractivity	TPSA	Lipinsiki's rule-of-five violation	Bioavailability score
1	Alpha-pinene	136.23	0	0	0	3	45.22	0	1	0.55
2	Beta-phellandrene	136.23	0	0	1	3.16	47.12	0	0	0.55
3	Beta-pinene	136.23	0	0	0	3	45.22	0	1	0.55
4	Beta-sitosterol	414.71	1	1	6	8.02	133.23	20.23	1	0.55
5	Beta-spathulene	202.34	0	0	0	4.19	66.67	0	1	0.55
6	Camphene	136.23	0	0	0	3	45.22	0	1	0.55
7	Carvacrol	150.22	1	1	1	2.82	48.01	20.23	0	0.55
8	Caryophyllene	204.35	0	0	0	4.73	68.78	0	1	0.55
9	D-Alpha-phellandrene	136.23	0	0	1	3.16	47.12	0	0	0.55
10	D-LImonene	136.23	0	0	1	3.31	47.12	0	0	0.55
11	Fisetin	286.24	4	6	1	2.28	76.01	111.13	0	0.55
12	Gallic-acid	170.12	4	5	1	0.5	39.47	97.99	0	0.56
13	Kaempferol	286.24	4	6	1	2.28	76.01	111.13	0	0.55
14	Myrcene	136.23	0	0	4	3.48	48.76	0	0	0.55
15	Myricetin	318.24	6	8	1	1.69	80.06	151.59	1	0.55
16	Perillaldehyde	150.22	0	1	2	2.49	47.32	17.07	0	0.55
17	Quercetin	302.24	5	7	1	1.99	78.03	131.36	0	0.55
18	Sobrerol	170.25	2	2	1	1.47	49.96	40.46	0	0.55
Control	Copanlisib	480.52	2	9	8	-0.26	135.06	142.01	1	0.55

The assessment of pharmacokinetic properties revealed that the compounds carvacrol, fisetin, gallic acid, kaempferol, perillaldehyde, quercetin, and sobrerol possess substantial absorption in the GIA tract. Conversely, beta-sitosterol, caryophyllene, fisetin, gallic acid, kaempferol, myricetin, and quercetin were found to lack permeability across the BBB. Notably, none of the examined compounds functioned as p-glycoprotein (p-gp) substrates, a vital efflux transporter associated with drug resistance.

Regarding interactions with specific cytochrome P450 (CYP) isoenzymes, carvacrol, fisetin, kaempferol, myricetin, and quercetin were identified as inhibitors of CYP1A2. Caryophyllene was established as an inhibitor of CYP2C19, while alpha-pinene, beta-pinene, beta-spathulene, camphene, caryophyllene, and D-limonene exhibited inhibition of CYP2C9. In addition, fisetin, kaempferol, and quercetin displayed inhibitory effects on CYP2D6, and fisetin, gallic acid, kaempferol, myricetin, and quercetin exhibited inhibition of CYP3A4. These findings underscore the diverse pharmacokinetic attributes and potential interactions of these compounds with essential drug-metabolizing enzymes. Table 3 offers a comprehensive insight into the pharmacokinetic characteristics of the phytochemicals. It emphasizes vital factors, such as their absorption and permeability across the BBB, whether they act as substrates for p-glycoprotein, and their interactions with various cytochrome P450 isoenzymes.

**Table 3:** Pharmacokinetic properties of the phytochemicals selected from Schinus molle GIA: gastrointestinal tract absorption; BBB: blood-brain barrier; p-gp: p-glycoprotein

S. No.	Phytochemicals	GIA	BBB permeant	p-gp substrate	CYP1A2 inhibitor	CYP2C19 Inhibitor	CYP2C9 inhibitor	CYP2D6 inhibitor	CYP3A4 inhibitor
1	Alpha-pinene	Low	Yes	No	No	No	Yes	No	No
2	Beta-phellandrene	Low	Yes	No	No	No	No	No	No
3	Beta-pinene	Low	Yes	No	No	No	Yes	No	No
4	Beta-sitosterol	Low	No	No	No	No	No	No	No
5	Beta-spathulene	Low	Yes	No	No	Yes	Yes	No	No
6	Camphene	Low	Yes	No	No	No	Yes	No	No
7	Carvacrol	High	Yes	No	Yes	No	No	No	No
8	Caryophyllene	Low	No	No	No	Yes	Yes	No	No
9	d-Alpha-phellandrene	Low	Yes	No	No	No	No	No	No
10	d-Limonene	Low	Yes	No	No	No	Yes	No	No
11	Fisetin	High	No	No	Yes	No	No	Yes	Yes
12	Gallic-acid	High	No	No	No	No	No	No	Yes
13	Kaempferol	High	No	No	Yes	No	No	Yes	Yes
14	Myrcene	Low	Yes	No	No	No	No	No	No
15	Myricetin	Low	No	No	Yes	No	No	No	Yes
16	Perillaldehyde	High	Yes	No	No	No	No	No	No
17	Quercetin	High	No	No	Yes	No	No	Yes	Yes
18	Sobrerol	High	Yes	No	No	No	No	No	No
Control	Copanlisib	Low	No	Yes	No	No	Yes	No	Yes

Molecular docking

**Figure 2:**
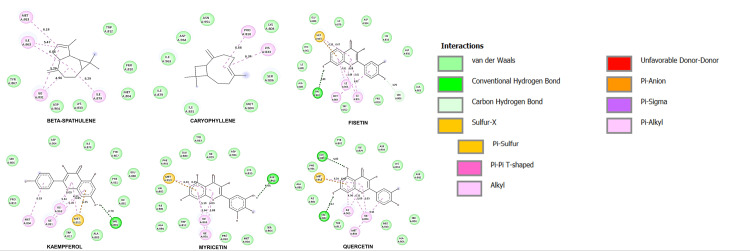
2D intermolecular interactions between the ligands and the amino acid of the target protein PI3K

Table 4 furnishes in-depth insights into the binding affinity and the intricate amino acid interactions between the bioactive compounds and PI3K. The recorded binding affinities of these compounds spanned from -5.2 to -8.3 kcal/mol. Notably, within this range, the compounds beta-spathulene, caryophyllene, fisetin, kaempferol, myricetin, and quercetin displayed affinities ranging from -6.5 to -8 kcal/mol. It was found that the control drug exhibited a binding affinity of -8.4 kcal/mol. Figure 2 provides the 2D docked structure of the ligands in conjunction with PI3K and also offers a deeper understanding of the ligand's binding orientations within the active site of PI3K with an affinity of -6.5 to -8 Kcal/mol and provides insights into their potential interactions with key amino acid residues in the binding pocket. These six compounds were selected for subsequent evaluations.

**Table 4:** Binding affinities and the interactions with amino acids between the bioactive compounds and PI3K NHB: number of hydrogen bond

S. No.	Chemical	Binding affintiy	NHB	Hydrogen bond	Carbon hydrogen / Pi-anion / Pi-sulfur / Pi-Pi stacked / Pi-alkyl bond	Van der Waals
1	Alpha-pinene	-5.4	0		Lys833, Ile831, Ile879, Ile963	Asp964, Asn951
2	Beta-phellandrene	-5.5	0		Ile879, Ile963, Ile831	Asp964, Tyr867, Leu838, Lys833, Phe961, Met953
3	Beta-pinene	-5.3	0		Ile879, Lys833, Ile831, Ile963	Asp964, Asn951
4	Beta-sitosterol	-8.3	0		Tyr867, Met953, Val882, Phe961, Ile831, Ile963, Met804, Ala805	Ile879, Asp964, Lys833, Pro810, Ser806, Lys807, Asp950, Lys890, Try812, Ile881, Ala885, Glu880
5	Beta-spathulene	-7.4	0		Met953, Ile963, Ile831, Ile879	Tyr867, Asp964, Lys833, Met804, Pro810, Trp812
6	Camphene	-5.2	0		Ile879, Ile831, Ile963	Lys833, Asp964, Met953, Met804
7	Carvacrol	-6.1	1	Asp964(4.01)	Ile831, Ile879, Ile963, Tyr867	Lys833, Phe961, Met953, Glu880, Ile881, Val882
8	Caryophyllene	-6.5	0		Pro810, Lys833	Met804, Ser806, Lys808, Asn951, Asp964, Ile963, Ile879, Ile831
9	d-Alpha-phellandrene	-5.9	0		Leu838, Ile831, Ile963, Ile879, Met953	Lys833, Tyr867, Asp964, Phe961
10	d-Limonene	-6.1	0		Ile831, Ile879, Ile963, Met953, Leu838	Phe961, Asp964, Tyr867, Lys833
11	Fisetin	-7.4	1	Val882(2.89)	Met953, Ile963, Ile831, Met804, Ser806	Ala805, Asp950, Lys833, Asp964, Ile879, Glu880, Phe961, Ile881, Ala885, Trp812, Pro810
12	Gallic-acid	-5.3	3	Arg277(6.58), Thr827(5.22,4.70)),Gly829(2.58,3.54)	Lys883	Glu826, Ile828, Ile830, Ile881, Glu880, Tyr787, Asp788, Leu791
13	Kaempferol	-7.5	1	Val882(2.70)	Met804, Ile831, Ile963, Met953	Pro810, Ser806, Asp964, Ile879, Tyr867, Glu880, Phe961, Ile881, Ala885, Trp812
14	Myrcene	-5.6	0		Val882, Ile881, Tyr867, Ile831, Lys833, Ile879, Ile963, Met953	Ala885, Phe961, Glu880, Asp964
15	Myricetin	-7.3	1	Asp950(4.15)	Met953, Ile963, Ile831	Ser806, Met804, Pro810, Trp812, Ala885, Ile881, Val882, Phe961, Glu880, Tyr867, Ile879, Asp964, Lys833
16	Perillaldehyde	-6	0		Met953, Ile831, Ile963, Ile879, Asp964	Phe961, Tyr867, lys833, Leu838, Asp841
17	Quercetin	-7.5	2	Glu880(5.00), Val882(3.12)	Met953, Ile963, Met804, Ile831	Ser806, Ala805, pro810, Trp812, Ile881, Ala885, Phe961, Tyr867, Ile879, Asp964, Lys833, Asp950
18	Sobrerol	-5.4	0		Ile879, Ile963, Ile831, Lys833	Tyr867, Asp964, Trp812, Met953, Thr887, Lys890, Met804
C	Copanlisib	-8.4	3	Asp836(4.30), Asn951(4.76), Val882(3.78)	Lys808, Asp964, Met953, Ile881, Met804, Asp950, Ser806	Lys833, Ile963, Ile879, Phe961, Glu880, Ala885, Ile831, Lys890, Trp812, Ala805, Lys807

Toxicity profile

Table 5 provides an overview of the toxicity profiles associated with the compounds. Among the six compounds evaluated, two compounds, namely, beta-spathulene and kaempferol, were devoid of any predicted toxicological endpoints according to our analysis conducted with ProTox-II. Moreover, these compounds were categorized under the acute toxicity class 5, which implies a low level of acute toxicity potential. The LD_50 _(lethal dose for 50% of the test population) values were determined to provide further insights. For beta-spathulene, LD_50_ was recorded as 4,690 mg/kg, while for kaempferol, it was 3,919 mg/kg. This analysis contributes to understanding the compounds' safety profiles and provides essential information for potential future therapeutic applications.

**Table 5:** Toxicity profiles of the selected phytochemicals LD_50_: Lethal dose 50

S. No.	Phytochemical	LD_50_ (mg/Kg)	Acute toxicity class	Hepatoxicity	Carcinogenicity	Immunotoxicity	Mutagenicity	Cytotoxicity
1	Beta-spathulene	4690	5	Inactive	Inactive	Inactive	Inactive	Inactive
2	Caryophyllene	5300	5	Inactive	Inactive	Active	Inactive	Inactive
3	Fisetin	159	3	Inactive	Active	Inactive	Inactive	Inactive
4	Kaempferol	3919	5	Inactive	Inactive	Inactive	Inactive	Inactive
5	Myricetin	159	3	Inactive	Active	Inactive	Active	Inactive
6	Quercetin	159	3	Inactive	Active	Inactive	Active	Inactive
Control	Copanlisib	2935	5	Inactive	Active	Active	Inactive	Inactive

Bioavailability radar and molecular target

**Figure 3:**
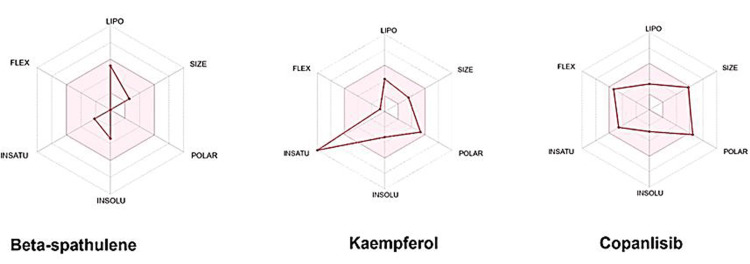
Radar plots from SwissADME depicting the oral bioavailability of the identified hits

**Figure 4:**
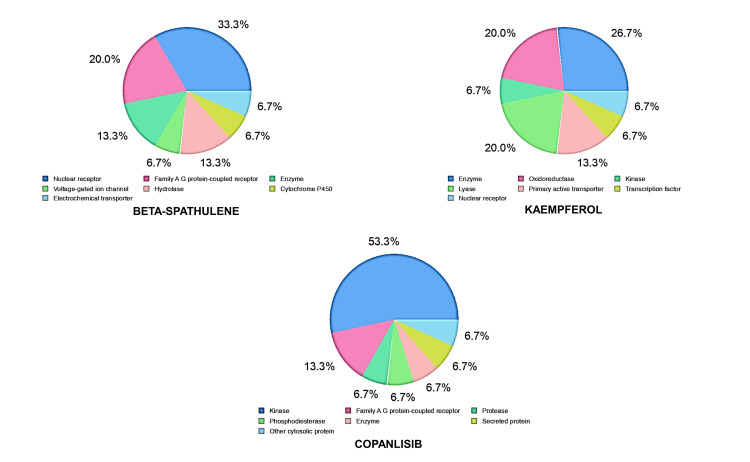
Predicted molecular targets of the identified leads, forecasting interactions with biological targets

Figure 3 portrays radar plots showcasing beta-spathulene and kaempferol. The plots spotlight the desirable range for each compound, depicted by the hue pink. Compounds within this pink radar plot sector signify a favorable outlook for oral bioavailability. Figure 4 elucidates the molecular targets affiliated with the identified hits. Analyzing these molecular targets holds pivotal importance in drug discovery and development, and comprehending the prospective therapeutic roles of these compounds might be undertaken. Molecular target analysis involves identifying and exploring specific proteins, enzymes, receptors, or other biomolecules with which a particular compound interacts.

## Discussion

Our* in silico *investigation aimed to explore the potential of phytochemicals from *S. molle* as inhibitors of the PI3K pathway in cancer. The selected compounds adhered to essential drug-likeness and pharmacokinetic criteria, setting the stage for further evaluation. Molecular docking analyses provided a comprehensive understanding of their binding interactions with the PI3K target, revealing promising candidates, such as beta-spathulene, caryophyllene, fisetin, kaempferol, myricetin, and quercetin, which exhibited a substantial affinity of -6.5 to -8 kcal/mol. ProTox-II investigations indicated that two compounds, beta-spathulene and kaempferol, showed no predicted toxicological endpoints. The bioavailability radar plot of these two identified leads possess oral bioavailability.

Yadav and Mohite conducted an intricate examination into the drug-likeness and pharmacokinetics of phytochemicals extracted from *Psidium guajava*. Utilizing the SwissADME web server, the study unveiled various phytochemicals from *Psidium guajava*, highlighting notable drug-like characteristics and favorable pharmacokinetic profiles [25]. Inspired by the valuable insights from Yadav and Mohite's drug-likeness exploration, our investigation focused on the phytochemicals reported from* S. molle, *employing the SwissADME web server. The outcomes of our study were consistent with the findings of the research carried out by Yadav and Mohite.

Muthiah *et al.* conducted a study on docking investigations to explore the interaction between phytochemicals and the GPR116 target in breast cancer. The observed significant affinity between the phytochemicals and the target protein suggested their potential as promising candidates for breast cancer treatment [26]. Our docking study results obtained from the interactions between the phytochemicals derived from *S. molle *and PI3K aligned with these observations. This outcome consistency further supports the notion of the therapeutic potential carried by these phytochemicals.

Roy et al. conducted a docking analysis to examine the interactions between the HER4/ErbB4 kinase and ERα with 44 compounds. They identified the compounds with the highest affinity, and their study aligns with our research, reinforcing our findings with a similar approach [22].

In their research, Kadri and Aouadi employed the bioavailability radar to analyze compounds' oral bioavailability [27]. Based on this research, we integrated the bioavailability radar plot as a valuable tool to appraise oral bioavailability. Our findings closely paralleled theirs, affirming the advantageous oral bioavailability characteristics intrinsic to the compounds. This alignment in outcomes validates our chosen methodology and accentuates the potential significance of the identified compounds as potential candidates harboring promising therapeutic attributes. The alignment of our findings with prior research reinforces the credibility of our approach. Furthermore, the results were found to be concordant with the control.

Limitations and future recommendations

Although our study marks a promising inception in developing potential anti-cancer compounds sourced from plants, it is imperative to acknowledge the accompanying limitations. The findings we have obtained in our study are primarily based on predictive models and computational analyses. While these results offer valuable insights and guidance, they should be considered a preliminary step in the research process. Therefore, experimental validation for identified leads is crucial as it provides concrete evidence regarding the effectiveness and safety of the compounds.

## Conclusions

This study pursued a computational exploration into the potential of phytochemicals derived from *S. molle* as inhibitors targeting PI3K in the context of cancer. Our investigative efforts yielded two prominent leads, beta-spathulene and kaempferol, exhibiting remarkable affinities toward PI3K. These compounds demonstrated favorable attributes related to drug-likeness, pharmacokinetics, and bioavailability and emerged as potential candidates for further exploration. Further experimental validation is essential to confirm the effectiveness of the identified leads in our study.
